# The Clinical and Laboratory Profiles of Immunocompetent Patients With Short-Duration Fever With Neutropenia in a Tertiary Care Hospital in Pune, India

**DOI:** 10.7759/cureus.34818

**Published:** 2023-02-09

**Authors:** Nikitha Nimmagadda, Kishor M Khillare, Prasanna Kumar Satpathy, Bharath S Gowda, Varun Narayana, Prashant Gopal, Srikanth Tripathy, Prachi V Athavale

**Affiliations:** 1 Internal Medicine, Dr. Dnyandeo Yashwantrao (DY) Patil Medical College, Hospital and Research Centre, Dr. DY Patil Vidyapeeth, Pune, IND; 2 Gastroenterology and Hepatology, Sapthagiri Institute of Medical Sciences and Research Centre, Bangalore, IND; 3 Internal Medicine, Mahavir Institute of Medical Sciences, Vikarabad, IND; 4 Gastroenterology, Dr. Dnyandeo Yashwantrao (DY) Patil Medical College, Hospital and Research Centre, Dr. DY Patil Vidyapeeth, Pune, IND; 5 Central Research Facility, Dr. Dnyandeo Yashwantrao (DY) Patil Medical College, Hospital and Research Centre, Dr. DY Patil Vidyapeeth, Pune, IND; 6 Microbiology, Dr. Dnyandeo Yashwantrao (DY) Patil Medical College, Hospital and Research Centre, Dr. DY Patil Vidyapeeth, Pune, IND

**Keywords:** c-reactive protein, antibiotic, headache, neutropenic infections, fever

## Abstract

Background

Management of a febrile patient is based on understanding the pathophysiology of an abnormal temperature and temperature regulation, impacts of fever, and its treatment. In the current study, we aimed to characterize and compare the epidemiological, etiologic, microbiological, serological, clinical, and outcome traits of febrile patients with acute neutropenia admitted to a tertiary care center in Western Maharashtra.

Methods

Adult patients with a history of fever of less than two weeks’ duration and without any immunosuppressive state were screened with predefined inclusion and exclusion criteria. General and demographic information (age and gender), and clinical examinations (type and duration of fever) were recorded. Biochemical, hematologic (total and differential cell counts), and immunologic measurements (rapid malaria, dengue, *Leptospira*, and viral hepatitis antigen antibodies) were performed. Data were analyzed using an appropriate statistical package.

Results

A total of 403 (214 males) young adults (aged: 29±11 years) with clinical presentation of fever were studied. The majority (n=361, 89.6%) had low-grade continuous fever with an average duration of 3±1 (mean±standard deviation (SD)) days. Headache and myalgia were the common symptoms present, and patients had an average hospital stay of 4±1 days. Dengue (55%) was the most common cause of febrile neutropenia, and all patients recovered well without antibiotics and granulocyte colony-stimulating factor. The mean C-reactive protein (CRP) level was 61.4±4.4 mg/L. CRP and procalcitonin (PCT) were directly correlated with the degree of neutropenia and inversely correlated with total leucocyte count (TLC).

Conclusions

It was highlighted from this study that antibiotics are not necessary for viral infections that have been diagnosed to stop the development of secondary bacterial infections. A clinician should be aware of “when not to use antibiotics,” or the world will soon have to deal with superbugs.

## Introduction

One of the most prevalent and concerning symptoms that medical professionals must evaluate is fever. The management of a febrile patient requires a thorough understanding of what constitutes a fever, the pathophysiology of temperature regulation, the beneficial and harmful impacts of fever, and its treatment.

Fever is defined as an increase in the temperature of the body that is more than the usual daily range and occurs in conjunction with a rise in the hypothalamic set point. Increased muscular tone and shivering are due to a rise in hypothalamic set point.

Normal body temperature follows a diurnal rhythm, rising in the afternoon and falling in the early morning with the range being 36.5°C to 37.5°C.

The concept of fever is also mentioned in human myth, art, and science. Physicians presented a thorough description of fever and its many patterns of presentation since the time of Hippocrates and, subsequently, during the Roman Empire. The development of a reliable thermometer by the Dutch instrument maker Gabriel Daniel Fahrenheit at the beginning of the 18th century sparked renewed interest in the connection between body temperature and illness [1].

Fever is one of the presenting symptoms in numerous illnesses, ranging from benign to potentially fatal diseases. Fever can be present together with other symptoms or signs that point to organ involvement. Pyrogens are substances that induce fever. Pyrogens can be exogenous, such as lipopolysaccharides, enterotoxins, and exotoxins, or endogenous, such as interleukins, tumor necrosis factor, and interferons [2].

In immunocompetent individuals, acute transitory neutropenia is primarily a benign and brief illness linked to an intercurrent viral infection, especially in patients who are afebrile. While a viral etiology is still frequently present in immunocompetent febrile individuals, severe bacterial infections (SBIs) should always be considered [3].

An absolute neutrophil count of less than 1,500 cells/mm^3^ is defined as neutropenia. It is classified based on severity. Due to transient bone marrow inhibition, viral infections frequently result in neutropenia. The common viruses associated are cytomegalovirus (CMV), hepatitis A virus (HAV), influenza virus, measles, and Epstein-Barr virus (EBV) [4].

Neutropenia can be brought on by any bacterial infection, but it is most frequently associated with rickettsia, pertussis, brucellosis, and salmonellosis infections. Neutropenia has also been linked to miliary tuberculosis. Bone marrow dysfunction is occasionally observed in disseminated histoplasmosis, and it is uncommon in fungal infections [5].

The major goal of the current study was to characterize and compare the epidemiological, etiologic, microbiological, serological, clinical, and outcome traits of febrile patients with acute neutropenia admitted in a tertiary care center in Western Maharashtra.

## Materials and methods

This prospective observational study was conducted at Dr. Dnyandeo Yashwantrao (DY) Patil Medical College, Hospital and Research Centre, Pimpri, Pune, from October 2020 to September 2022. Adult patients without any immunocompromising medications or illness presenting with a fever of less than two weeks’ duration were screened. Patients with prior exposure to antibiotics, chemotherapy, or radiotherapy, those who were taking drugs causing neutropenia (such as carbimazole, clozapine, dapsone, and penicillin G), and pregnant women were excluded.

Eligible participants who agreed to participate signed informed written consent. The study was approved by the Institutional Ethics Sub-Committee (IESC/PGS/2020/05).

The investigations that were carried out based on the clinical diagnosis were rapid malaria test by an immune chromatographic assay based on antigen detection using Advantage MAL Card, urine culture, blood culture, and dengue serology (nonstructural antigen 1 (NS1), Dengue immunoglobulin G (IgG), and Dengue immunoglobulin M (IgM)) by an immune chromatographic method using Quanti Card and microwell enzyme-linked immunosorbent assay Widal test (slide and tube agglutination method using TYDAL kit).

Two 20 mL of blood samples were collected from two separate sites at one-hour intervals under all aseptic precautions before the initiation of antimicrobial therapy. This was inoculated into BACTEC (Becton, Dickinson and Company, Franklin Lakes, USA)-specific blood culture bottles and immediately sent to the microbiology laboratory for culture. A separate blood sample was also collected for immunological studies such as rapid malaria, dengue, *Leptospira*, and viral hepatitis serological testing.

Patients were thoroughly clinically evaluated as per a proforma. Blood investigations such as complete hemogram, platelet, erythrocyte sedimentation rate, peripheral blood smear for malarial parasites, rapid malaria, and liver function test (LFT) were carried out based on the clinical diagnosis.

Investigation results are correlated with clinical probabilities. The results were analyzed statistically using Statistical Package for the Social Sciences (SPSS) version 22.0 (IBM SPSS Statistics, Armonk, NY, USA). The studies were carried out after institutional ethical committee clearance.

Statistical methods

The following statistical software were used for the analysis: SAS version 9.2 (SAS Institute Inc., Cary, NC, USA), SPSS version 22.0, Stata version 10.1 (StataCorp LLC, College Station, TX, USA), MedCalc version 9.0.1 (MedCalc Software, Ostend, Belgium), Systat version 12.0 (Systat Software, Inc., San Jose, CA, USA), and R environment version 2.11.1 (RStudio, Boston, MA, USA).

Continuous measurement results were presented as mean±standard deviation (SD) (minimum-maximum), while categorical measurement results were presented as numbers (percentages). At the 5% level, significance is determined. The significance of study parameters among three or more groups of patients has been determined using analysis of variance (ANOVA) and chi-square/Fisher’s exact test. The significance of study parameters on a categorical scale between two or more groups has been determined using the exact test.

## Results

A total of 403 patients with a clinical presentation of fever were enrolled in the study. Patients had an average age of 29±11 years. The majority of the patients (41.4%) belonged to the 15-24 years age group, followed by 25-34 years (32%) and 35-44 years (16.1%). Of the patients, 10.5% were more than 45 years of age. We observed a slight male predominance (n=403, 53.1% males and 46.9% females) (Table 1).

**Table 1:** General and demographic profile of the patients (N=403) Data are presented as number (%) or mean±SD. SD: standard deviation

				Frequency	Percentage	
Age (in years)		15-24		167	41.4	
		25-34		129	32	
		35-44		65	16.1	
		45-54		26	6.5	
		55-64		10	2.5	
		>64		6	1.5	
		Total		403	100	
Mean±SD		29.4243±11.25401				
			Frequency			Percentage
Gender	Female		189			46.9
	Male		214			53.1
	Total		403			100

Low-grade continuous fever was recorded in 361 (89.6%) patients, 3.5% had stepladder fever, and 3% had high-grade intermittent fever. Headache and body ache were commonly observed symptoms (Table 2), and the mean duration of fever was 3±1 days.

**Table 2:** Types of fever and clinical presentation (N=403) Data were presented as number (%) or mean±SD. SD: standard deviation

			Number of cases	Percentage	
Fever type and symptoms	Low-grade, continuous		361	89.6	
	Nonspecific		14	3.5	
	Stepladder		14	3.5	
	High-grade, intermittent		12	3	
	High-grade, continuous		2	0.5	
Chill		12			3
Cold		132			32.8
Headache		356			88.3
Body ache		356			88.3
Loose stool		14			3.5
Myalgia		223			55.3
Arthralgia		226			56.1
Rash		237			58.8
Icterus		0			0

The mean total leucocyte count (TLC) at the time of admission was 4,999/µL (normal: 4,000-11,000/µL), with a standard deviation of 3,317.69, while the mean TLC at the time of discharge was 6,396.52/µL, with a standard deviation of 4,276.83. The absolute neutrophil count (ANC) at the time of admission was 2,334/µL (normal: 1,500-7,000/µL), with a standard deviation of 1,750.77, while the ANC at the time of discharge was 3,717.91/µL, with a standard deviation of 2,898.23. The platelet count at the time of admission was 107,352/µL (normal: 150,000-400,000/µL), with a standard deviation of 89,981, while the platelet count at the time of discharge was 152,091/µL, with a standard deviation of 69,849. Thus TLC, ANC, and platelet counts were improved after supportive treatment (Table 3).

**Table 3:** Mean TLC, neutrophils, and platelet values (N=403) Data were presented as mean±SD, and minimum and maximum. TLC: total leucocyte count, ANC: absolute neutrophil count, SD: standard deviation

	Minimum	Maximum	Mean	SD
TLC (/µL)				
Admission	1,000.00	24,400.00	4,999.2	3,317.6
Discharge	3,560.00	70,000.00	6,396.5	4,276.8
ANC (/µL)				
Admission	400.00	11,690.00	2,334.0	1,750.7
Discharge	504.00	49,700.00	3,717.9	2,898.2
Platelet (/µL)				
Admission	5,000.00	773,000.00	107,352.3	89,981.1
Discharge	101,000.00	577,000.00	152,091.8	69,849.5

The mean±SD hospital stay was 4±1 days for these patients. The mean±SD C-reactive protein (CRP) level was 61.4±44.41 mg/dL (normal: <6 mg/dL) with a range of 2-281.

Of 403 patients, only 28 (6.9%) normal were on antimicrobial treatment, while 375 (93.1%) normal had no antibiotic coverage. The final diagnosis revealed that 221 (55%) patients had dengue fever (DF), followed by 135 (33%) patients with viral fever (Table 4). On grading for neutropenia, 26 (6%) patients had severe neutropenia, while the majority of patients had normal neutrophil values.

**Table 4:** Diagnosis and treatment Data were presented as number (%). COVID-19: coronavirus disease 2019

		Frequency	Percentage
Diagnosis	Leptospirosis	2	0.5
	Chikungunya	5	1.2
	Malaria	12	3
	COVID-19 fever	14	3.5
	Enteric fever	14	3.5
	Viral fever	135	33.5
	Dengue fever	221	54.8
	Total	403	100
Antibiotics coverage	Yes	28	6.9
	No	375	93.1
	Total	403	100

Among various investigations performed on the enrolled patients, the following results were observed. In dengue serology testing by enzyme-linked immunosorbent assay method using the microwell kit, 45.2% of the patients tested negative for dengue, 23.6% were IgM-positive, 22.6% were NS1-positive, 7.2% were IgG-positive, 0.7% were NS1- and IgM-positive, and 0.7% were IgM- and IgG-positive (Table 5).

**Table 5:** Distribution according to dengue serology NS1: nonstructural antigen 1, IgM: immunoglobulin M, IgG: immunoglobulin G, +: positive, -: negative

		Frequency	Percentage
Dengue serology	IgM+ and IgG+	3	0.7
	NS1+ and IgM+	3	0.7
	IgG+	29	7.2
	NS1+	91	22.6
	IgM+	95	23.6
	-	182	45.2
	Total	403	100

In a rapid malaria test by immunochromatography assay using Advantage MAL Card, 97% of the patients had a negative rapid test, 2% were positive for *Plasmodium falciparum*, and 1% were positive for *Plasmodium vivax*.

In the Widal test by slide and tube agglutination using TYDAL kit results, 97.8% of the patients were Widal-negative and only 2.2% were WIDAL-positive.

Blood culture was performed by automated blood method (BACT/ALERT 3D), and out of 403 enrolled patients, 96.5% had negative blood culture results, while only 3.5% were positive for blood culture, and *Salmonella typhi* was isolated from these patients.

Urine culture and sensitivity testing were performed on mid-stream urine samples, and 99% of the patients had negative urine culture results, while from 0.5% of patients, *S. typhi* was isolated, and *Klebsiella pneumoniae* and *Escherichia coli* were isolated from 0.2% each.

In stool culture results, 99.5% of the patients had negative stool culture results, while 0.2% were positive for *S. typhi*, and 0.2% were positive for *E. coli*.

On the statistical analysis of the relation between grades of neutropenia and duration of hospital stay, all values were found to be nonsignificant (>0.001). CRP levels were found to be significant, except in moderate cases versus severe cases. Procalcitonin (PCT) values were found to be significant, except for moderate versus severe and normal versus mild cases (Table 6). When we checked the associations of TLC levels with inflammatory markers, CRP and PCT were found to be inversely associated with correlation coefficients of -0.579 and -0.472, respectively (Table 7).

**Table 6:** Association between the severity of neutropenia and the mean duration of hospital stay, mean CRP, and mean PCT The tests applied were one-way ANOVA. * indicates Bonferroni test (post hoc test). CRP: C-reactive protein, PCT: procalcitonin, ANC: absolute neutrophil count, ANOVA: analysis of variance, SD: standard deviation, NS: not significant, Sig.: significant

ANC (/µL)		Number	Mean	SD	p-value*					
					Normal versus mild	Normal versus moderate	Normal versus severe	Mild versus moderate	Mild versus severe	Moderate versus severe
Hospital stays	>1,500 (normal)	253	4.1383	1.69284	1.000 (NS)	0.126 (NS)	1.000 (NS)	1.000 (NS)	1.000 (NS)	0.984 (NS)
	1,000-1,500 (mild)	79	4.3418	1.83894						
	500-1,000 (moderate)	45	4.7778	1.53577						
	<500 (severe)	26	4.1923	1.67378						
	Total	403	4.2531	1.71051						
p-value	0.132 (NS)									
CRP	>1,500 (normal)	253	41.0593	26.92804	0.001 (Sig.)	0.001 (Sig.)	0.001 (Sig.)	0.001 (Sig.)	0.001 (Sig.)	0.195 (NS)
	1,000-1,500 (mild)	79	68.8481	27.07070						
	500-1,000 (moderate)	45	119.5778	45.77588						
	<500 (severe)	26	136.0385	46.73284						
	Total	403	61.4020	44.41160						
p-value	0.001 (Sig.)									
PCT	>1,500 (normal)	253	2.5557	2.73902	0.259 (NS)	0.001 (Sig.)	0.001 (Sig.)	0.001 (Sig.)	0.001 (Sig.)	0.694 (NS)
	1,000-1,500 (mild)	79	5.8835	8.13204						
	500-1,000 (moderate)	45	21.1089	29.59253						
	<500 (severe)	26	26.0500	27.46330						
	Total	402	6.8061	14.80401						
p-value	0.001 (Sig.)									

**Table 7:** Correlation between TLC and hospital stay, CRP, and PCT **Correlation is significant at the 0.01 level (two-tailed). TLC: total leukocyte count, CRP: C-reactive protein, PCT: procalcitonin, Sig.: significant

		Hospital stays	CRP	Procalcitonin
TLC	Pearson’s correlation	-0.037	-0.579**	-0.472**
	Sig. (two-tailed)	0.465	0.000 (Sig.)	0.000 (Sig.)

## Discussion

A total of 403 immunocompetent patients with short-duration fever were evaluated in our study. A detailed history, clinical examination, and hematologic, biochemical, microbiological, and serological tests were done to confirm the diagnosis. Data were collected as per proforma, compiled, and tabulated in observation.

In the present study, the majority of the participants belonged to the age group of 15-24 years with male (53.1%) preponderance. This can be explained by the increased correlation between males’ outdoor occupational exposure to mosquitoes and the transmission of vector-borne diseases [6,7].

Regarding age distribution in patients, our study findings are consistent with those of a study conducted at a tertiary healthcare institute in Uttarakhand, India, to study etiological patterns in an epidemic of acute febrile illness during monsoon, and more patients were in the age group of 21-30 years (25.5%), followed by the age group of 31-40 years (20.7%) [8]. However, our study findings related to age distribution are not in agreement with the study conducted by Salh et al. [9], in which the majority of patients were in the age groups of 31-40 years (30.3%) and 21-30 years (23%).

Low-grade continuous fever was the most common type of fever seen and observed in 89.6% of patients. Prodromal symptoms such as headache, arthralgia, and myalgia were seen in the majority of patients. In one study [10], the most frequent presenting symptoms were headache (74.3%), myalgia (56.5%), and nausea and vomiting (62.2%). Chills and rigors (48.1%), malaise (46.8%), and myalgia (23.2%) were shown to be the most frequently related symptoms to febrile illness in a different study from Cambodia [11].

Our study results were consistent with many studies conducted across India and other tropical countries.

In a multicentric study conducted in Kerala, dengue was the primary factor in 576 (43.5%) cases. The precise etiology could not be determined in 396 (29.9%) cases. Leptospirosis, enteric fever, malaria, respiratory tract infection, urinary tract infection, and typhus were some more causes, listed in that order [12].

Our study findings are not in agreement with those of a tertiary care center in Central India. In this study, out of 270 patients, 127 (47%) had scrub typhus, 33 (12%) had malaria, 47 (17.40%) had dengue, 12 (4%) had enteric fever, five (2%) had leptospirosis, 18 (6.66%) had no diagnosis, and 28 (10.37%) had other infections, including viral, urinary tract infection, upper and lower respiratory tract infection, and acute gastroenteritis [13]. Thus, geographical variation is one of the important factors in the causes of febrile illness in tropical countries.

A study conducted at Gorakhpur, India, revealed that the spotted fever group (SFG) and typhus group (TG) rickettsia are endemic in this region, and positive serological tests are reported for these illnesses. IgM and IgG antibodies against SFG were detected in 13.6% (40/294) and 36.7% (108/294) of individuals, respectively. IgM and IgG antibodies to TG were detected in 7.1% (21/294) and 15.3% (45/294) of individuals, respectively [14].

Procalcitonin was elevated in patients with bacterial infections. Out of 403 patients, 150 had neutropenia, of which 79 had mild neutropenia, 45 had moderate neutropenia, and 26 had severe neutropenia. Dengue was the most common cause of febrile neutropenia, and all patients recovered well without antibiotics and granulocyte colony-stimulating factor. Higher levels of CRP and procalcitonin were seen in neutropenic patients as it indicates severe infection and inflammatory response.

In a review study conducted on 55 publications with 265 biomarker entries, the most recently evaluated biomarkers include well-known biomarkers such as procalcitonin and C-reactive protein [15].

Our study findings are also consistent with an observational study conducted at a tertiary care hospital in North India, which concluded that PCT, CRP, TLC, and neutrophil/lymphocyte count ratio (NLCR) are quick and reliable biomarkers that can be used to determine whether febrile children are more likely to develop bacteremia or serious bacterial infections [16].

The use of biomarkers such as procalcitonin (PCT) and C-reactive protein (CRP) as guidelines for prescribing antibiotics in tropical countries, as well as high-income nations, has decreased antibiotic use while protecting patient safety [17].

The limitations of this study are its small sample size, bias in patient selection, endemicity of tropical infections in the study area, and the study being a single-center study.

## Conclusions

The most common cause of short-duration fever in patients under 30 years old was dengue. More than one-third of the patients had neutropenia. In all patients with short-duration fever, it was benign, temporary, and self-limiting. The majority of patients received symptomatic care without the use of antibiotics. Patients with short-duration fever without any clinical symptoms of bacterial infection and without hemodynamic compromise are to be treated with acetaminophen. Granulocyte colony-stimulating factor has no role in immunocompetent patients with febrile neutropenia. The usage of antibiotics prophylactically to prevent secondary bacterial infection is to be avoided.
